# Supplementary material for: The Link between Sleep Insufficiency and Self-Injury among In-School Adolescents: Findings from a Cross-Sectional Survey of Multi-Type Schools in Huangpu District of Shanghai, China
Source: Int J Environ Res Public Health. 2022 Nov 24;19(23):15595. doi: 10.3390/ijerph192315595 (PMC9740407; doi:10.3390/ijerph192315595)
Supplement: Supplementary file 1 [file ijerph-19-15595-s001.zip › ijerph-1990578-supplementary.pdf]

Supplementary Table S1. Correlation of Self-injury and sleep insufficiency among male and female students using multivariate logistic regression with the standard error of school type clustered.

	**Male**			**Female**		
	**AOR**	**95% CI**	***p***	**AOR**	**95% CI**	***p***
Sleep insufficiency	0.79	0.51–1.23	0.295	1.73	1.28–2.34	<0.001
School type						
(Key school)						
Ordinary school	3.19	0.51–19.87	0.214	0.93	0.32–2.67	0.893
Vocational school	1.07	1.01–1.15	0.034	1.38	1.09–1.74	0.008
Age	1.03	0.59–1.80	0.919	0.85	0.59–1.21	0.362
Ethnicity (Han)						
Minority	-	-	-	0.15	0.07–0.33	<0.001
Living with both parents						
Yes	0.83	0.32–2.13	0.691	1.04	0.43–2.53	0.926
School performance						
Average vs. worse off	0.69	0.24–1.99	0.491	0.26	0.07–0.89	0.032
Better off vs. worse off	0.39	0.20–0.76	0.006	0.86	0.73–1.00	0.056
Self-rated weight						
(Average)						
Lower than average	1.28	0.71–2.28	0.409	0.86	0.25–2.95	0.807
Higher than average	0.75	0.33–1.73	0.504	1.60	0.79–3.25	0.190
Smoking						
Yes	2.96	2.50–3.50	<0.001	1.23	0.83–1.81	0.307
Drinking						
Yes	1.02	0.84–1.24	0.822	1.68	0.78–3.61	0.184
Physical active						
Yes	1.51	0.51–4.49	0.457	1.13	0.77–1.66	0.537
Playing video game						
Over 4 hours	0.84	0.33–2.10	0.702	0.78	0.58–1.05	0.098
Bully Experience						
Moderate vs. None	2.32	1.88–2.87	<0.001	2.55	1.07–6.08	0.035
Severe vs. None	3.41	1.21–9.58	0.020	2.66	1.00–7.07	0.049
Mental Problem						
Moderate vs. Light	1.87	0.58–5.97	0.292	1.63	0.76–3.49	0.206
Severe vs. Light	4.19	2.67–6.57	<0.001	3.15	1.23–8.06	0.017

Note：No significant interactions between school types and sleep insufficiency were detected.

Supplementary Table S2. Correlation of Self-injury and sleep insufficiency (using 7 h as cut-off point) among male and female students using multivariate logistic regression with the standard error of school type clustered.

	**Total**			**Male**			**Female**		
	**AOR**	**95% CI**	***p***	**AOR**	**95% CI**	***p***	**AOR**	**95% CI**	***p***
Sleep insufficiency1.51	1.51	1.44–1.57	<0.001	2.11	1.88–2.38	<0.001	1.22	1.01–1.47	0.041
Sex	1.55	1.06–2.28	0.024	-	-	-	-	-	-
School type									
(Key school)									
Ordinary school	1.10	0.54–2.24	0.786	2.89	0.59–14.18	0.191	0.45	0.07–2.75	0.390
Vocational school	1.42	1.26–1.60	<0.001	1.29	1.00–1.67	0.054	1.59	1.27–1.99	<0.001
Interactions									
*Ordinary school * Sleep insufficiency*	3.30	2.59–4.22	<0.001	2.34	2.08–2.63	<0.001	3.61	1.93–6.75	<0.001
*Vocational school * Sleep insufficiency*	0.84	0.80–0.89	<0.001	1.00	0.77–1.31	0.999	0.63	0.54–0.75	<0.001
Age	0.87	0.68–1.11	0.258	0.97	0.57–1.64	0.898	0.79	0.51–1.23	0.305
Ethnicity (Han)									
Minority	0.91	0.32–2.60	0.856	-	-	-	0.26	0.07–1.03	0.054
Living with both parents									
Yes	0.90	0.66–1.21	0.476	0.91	0.37–2.22	0.835	0.95	0.44–2.04	0.899
School performance									
Average vs. worse off	0.47	0.41–0.53	<0.001	0.74	0.25–2.21	0.586	0.29	0.09–0.92	0.035
Better off vs. worse off	0.75	0.68–0.82	<0.001	0.48	0.30–0.76	0.002	0.99	0.78–1.27	0.981
Self-rated weight (Average)									
Lower than average	1.21	0.75–1.97	0.436	1.26	0.67–2.37	0.468	0.88	0.31–2.50	0.805
Higher than average	1.12	0.84–1.49	0.455	0.78	0.34–1.80	0.556	1.60	0.82–3.13	0.165
Smoking									
Yes	2.01	1.55–2.61	<0.001	2.92	2.24–3.80	<0.001	1.13	0.75–1.70	0.554
Drinking									
Yes	1.22	0.91–1.63	0.176	1.00	0.79–1.26	0.998	1.62	0.75–3.48	0.220
Physical active									
Yes	1.17	0.58–2.35	0.656	1.51	0.45–5.08	0.507	1.12	0.73–1.72	0.600

Playing video game									
Over 4 hours	0.84	0.57–1.23	0.370	0.84	0.35–2.01	0.700	0.81	0.63–1.03	0.089
Bully Experience									
Moderate vs. None	2.26	1.45–3.53	<0.001	2.25	1.96–2.58	<0.001	2.54	1.05–6.12	0.038
Severe vs. None	2.90	1.04–8.09	0.041	3.36	1.14–9.97	0.029	2.63	0.97–7.12	0.057
Mental Problem									
Moderate vs. Light	1.64	0.74–3.65	0.227	1.58	0.38–6.55	0.530	1.48	0.60–3.65	0.390
Severe vs. Light	3.44	1.77–6.66	<0.001	3.73	2.38–5.84	<0.001	2.97	0.95–9.31	0.062
